# Nuclear Ep-ICD Expression Is a Predictor of Poor Prognosis in “Low Risk” Prostate Adenocarcinomas

**DOI:** 10.1371/journal.pone.0107586

**Published:** 2015-02-19

**Authors:** Jasmeet Assi, Gunjan Srivastava, Ajay Matta, Christina MacMillan, Ranju Ralhan, Paul G. Walfish

**Affiliations:** 1 Alex and Simona Shnaider Laboratory in Molecular Oncology, Department of Pathology and Laboratory Medicine, Mount Sinai Hospital, 600 University Avenue, Toronto, Ontario, Canada; 2 Department of Pathology and Laboratory Medicine, Mount Sinai Hospital, Toronto, Ontario, Canada; 3 Department of Laboratory Medicine and Pathobiology, University of Toronto, Toronto, Ontario, Canada; 4 Joseph and Mildred Sonshine Family Centre for Head and Neck Diseases, Mount Sinai Hospital, Toronto, Ontario, Canada; 5 Department of Medicine, Endocrine Division, Mount Sinai Hospital and University of Toronto, Toronto, Ontario, Canada; 6 Department of Otolaryngology—Head and Neck Surgery, Mount Sinai Hospital, Toronto, Ontario, Canada; 7 Department of Otolaryngology—Head and Neck Surgery, University of Toronto, Toronto, Ontario, Canada; University of Kentucky College of Medicine, UNITED STATES

## Abstract

**Introduction:**

Molecular markers for predicting prostate cancer (PCa) that would have poor prognosis are urgently needed for a more personalized treatment for patients. Regulated intramembrane proteolysis of Epithelial cell adhesion molecule results in shedding of the extracellular domain (EpEx) and release of its intracellular domain (Ep-ICD) which triggers oncogenic signaling and might correlate to tumor aggressiveness. This study aimed to explore the potential of Ep-ICD and EpEx to identify PCa that have poor prognosis.

**Methods:**

Immunohistochemical analysis of Ep-ICD and EpEx was carried out in normal prostate tissues (n = 100), benign prostate hyperplasia (BPH, n = 83), and prostate cancer (n = 249) using domain specific antibodies. The expression of Ep-ICD and EpEx was correlated with clinico- pathological parameters and disease free survival (DFS).

**Results:**

Reduced expression of nuclear Ep-ICD and membrane EpEx was observed in PCa in comparison with BPH and normal prostate tissues (p = 0.006, p < 0.001 respectively). For patients who had PCa with Gleason Score less than 7, preserved nuclear Ep-ICD emerged as the most significant marker in multivariate analysis for prolonged DFS, where these patients did not have recurrence during follow up of up to 12 years (p = 0.001).

**Conclusion:**

Reduced expression of nuclear Ep-ICD was associated with shorter disease free survival in patients with a Gleason Score less than 7 and may be useful in identifying patients likely to have aggressive tumors with poor prognosis. Furthermore, nuclear Ep-ICD can differentiate between normal and prostate cancer tissues for ambiguous cases.

## Introduction

Prostate cancer (PCa) is the second most common cancer in the world, with an estimated 900,000 cases and 258,000 deaths in 2008 [1]. The United States will have an estimated 239,000 new cases, and 29,700 deaths in 2013 alone [2]. The ideal treatment for PCa continues to be a challenge for oncologists worldwide. There are curative treatments for PCa however, these are associated with increased patient morbidity; some of these patients are over-treated while others are under-treated. The incidence of PCa continues to rise with increased use of the screening tool, prostate specific antigen (PSA) resulting in an increase in indolent tumors that are managed by active surveillance, where patients would get biopsied periodically to detect disease progression [3]. Management of PCa relies heavily on a variety of factors namely, physical examination, PSA level, Gleason score (GS), clinical stage, tumor extent, invasion and imaging. Even with these clinical factors, prognosis is hard to define. Usually the size of tumor and appearance under microscope would mandate the patients’ treatment; some patients with good prognosis get the same treatment as patients with poor prognosis leading to under- or over- treatment. Furthermore, some PCa cases have diagnostic uncertainty where the pathology reports state “suspicious for cancer” [4]. The patients with these diagnoses are usually sent for a repeat biopsy, causing more distress. Thus, there is an unmet need for newer and better diagnostic and prognostic markers for more effective disease management.

Epithelial cell adhesion molecule (EpCAM) has been widely explored as an epithelial cancer antigen [5]. It is a glycosylated, 30- to 40-kDa type I membrane protein, expressed in several human epithelial tissues, and overexpressed in cancers as well as in progenitors and stem cells [5–9]. EpCAM is comprised of an extracellular domain (EpEx) with epidermal growth factor (EGF) and thyroglobulin repeat-like domains, a single transmembrane domain, and a short 26-amino acid intracellular domain called Ep-ICD. In normal cells, this full length EpCAM protein is sequestered in tight junctions and therefore less accessible to antibodies, whereas in cancer cells it is homogeneously distributed on the cancer cell surface and has been explored as a surface-binding site for therapeutic antibodies. EpCAM is expressed in majority of human epithelial cancers, including breast, colon, gastric, head and neck, prostate, pancreas, ovarian and lung cancer and is one of the most widely investigated protein for its diagnostic and therapeutic potential [10–13]. Increased EpCAM expression is a poor prognostic marker in breast and gall bladder cancers [14,15], while it is associated with favorable prognosis in colorectal and gastric cancers [16–19]. This paradoxical association of EpCAM expression with prognosis in different cancers may be explained by the functional studies of EpCAM biology using in vitro and in vivo cancer models [20]. Taken together these studies suggest that the impact of EpCAM expression in human cancers is likely to be context-dependent. EpCAM expression based assay is the only FDA-approved test widely used to detect circulating tumor cells in breast cancer [21].

EpCAM-targeted molecular therapies are being intensely pursued for several cancers including breast, ovarian, gastric and lung cancer [22,23]. EpCAM expression has been used to predict response to anti-EpCAM antibodies in breast cancer patients [22,24,25]. Surprisingly, clinical trials of anti-EpCAM antibodies targeting the EpEx domain have shown limited efficacy in cancer therapy and its negative prognostic potential for survival of cancer patients remains unclear [26–30]. This might be explained by the recently unraveled mode of activation of EpCAM oncogenic signaling by proteolysis and the potential of Ep-ICD in triggering more aggressive oncogenesis [19]. Regulated intra-membrane proteolysis of EpCAM results in shedding of EpEx and release of Ep-ICD into the cytoplasm, nuclear translocation and activation of oncogenic signaling [19]. Previously, we reported accumulation of Ep-ICD is frequently detected in ten epithelial cancers, including breast and prostate; in thyroid carcinomas nuclear Ep-ICD accumulation predicted poor prognosis [19,31].

The aim of this study was to evaluate the prognostic utility of Ep-CAM by characterizing the subcellular expression of Ep-ICD and membranous expression of EpEx in PCa using immunohistochemistry and correlating with clinic-pathological parameters and the follow up of patients. This would help investigate its potential to predict aggressive tumors that may aid in better management of patients.

## Materials and Methods

### Patients

**Fig 1:**
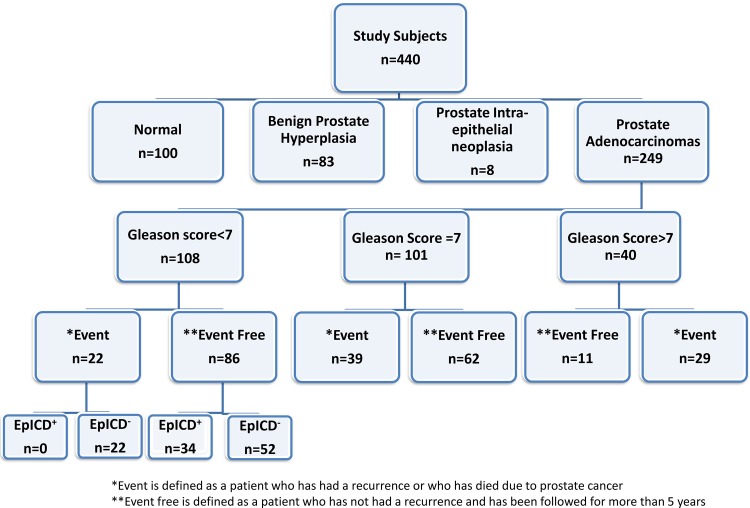
Study scheme of patient cohort. Patient cohort details are listed in a hierarchy manner.

This retrospective study of biomarkers using the PCa patients’ archived tissue blocks and their anonymized clinical data was approved by the Mount Sinai Hospital Research Ethics Board, Toronto, Canada; and have waived the need for consent. Patients who were considered to have a normal prostate had a diagnosis “negative for malignancy” that remained unchanged for the next 5 years with biopsies in between. These patients were required to have biopsy due to an increase in PSA. An aggressive cancer was considered if a patient had a biochemical recurrence as defined by the National Comprehensive Cancer Network (NCCN), distal metastasis, or died due to PCa; these were also considered as the clinical end points. All the tissues were re-reviewed by the pathologist (CM). These sections were chosen based on the highest score given by the pathologist on that specific tissue. Patients in this cohort had radical prostatectomy (RP, n = 101) or radiation therapy (RT, n = 143) and were free of disease after treatment. Patients considered to have a biochemical recurrence in our study cohort were patients that had an increase in PSA by 2 ng/ml after RT. Patients who had radical prostatectomy considered to have a biochemical recurrence were those that had a rise in PSA by 0.2 ng/ml on 2 occasions during follow-up. The schematic illustration of study design is given in Fig. 1. The inclusion criteria used in this study were based on PCa patients having an event or at least 5 years of follow-up without an event. Patients were excluded if there was incomplete follow-up information (less than 5 years) or if they were on active surveillance. Patients were followed up for up to 12 years.

### Ep-ICD / EpEx Immunohistochemistry

Serial formalin fixed and paraffin embedded (FFPE) tissue sections (4 μm thickness) were deparaffinized in xylene, hydrated through graded alcohol series, antigen was retrieved by microwave treatment, endogenous peroxidase activity was blocked and non-specific binding was blocked using normal horse (EpEx) or normal goat (Ep-ICD) serum (10%) as described earlier [32]. The sections were incubated with the mouse monoclonal antibody against EpEx (MOC-31, AbD Serotec, Oxford, UK) or rabbit anti-human Ep-ICD monoclonal antibody (Epitomics Inc., Burlingame, CA) for 1h. [19,33]. Slides were incubated with biotinylated secondary antibody for 20 min., followed by VECTASTAIN Elite ABC reagent (Vector labs, Burlingame, CA) using diaminobenzidine as the chromogen. Slides were washed with Tris-buffered saline (TBS, 0.1M, pH = 7.4), 3–5 times after every step. Sections were counterstained with Mayer's hematoxylin. In the negative control tissue sections, the primary antibody was replaced by isotype-specific non-immune mouse/rabbit IgG. The sections were evaluated by light microscopic examination.

### Evaluation of IHC Staining

IHC scoring was performed under supervision of the pathologist (CM) by two researchers (JA and GS) who were blinded to the clinical outcome. Immunopositive staining was evaluated in the most aggressive areas of tissue sections as described earlier [32]. Nuclear and cytoplasmic Ep-ICD, and membranous EpEx expression were evaluated independently in tumor cell nucleus, cytoplasm and membrane based on the intensity and percentage of positive staining. These sections were scored as follows: 0, < 10% cells; 1, 10–30% cells; 2, 31–50% cells; 3, 51–70% cells; and 4, > 70% cells showed immunoreactivity. Sections were also scored semi-quantitatively on the basis of intensity as follows: 0 = none; 1 = mild; 2 = moderate; and 3 = intense. Finally, a total score (ranging from 0 to 7) was obtained by adding the scores of percentage positivity and intensity for each of the cancer and non-cancerous tissue sections [32].

### Statistical Analysis

The IHC data were subjected to statistical analysis using SPSS 21.0 software (SPSS, Chicago, IL) and GraphPad Prism 5.0 software (GraphPad Software, La Jolla, CA). Scatter plots were used to determine the distribution of total score of cytoplasmic or nuclear Ep-ICD and EpEx expression in all tissues examined. p value < 0.05 was considered significant for statistical analysis [32]. Based on receiver operating curve analysis, the cut-offs of IHC score for preserved expression of cytoplasmic Ep-ICD and nuclear Ep-ICD were ≥ 4.0, and for membrane EpEx were ≥ 3.0 for further analysis. Expression data were analyzed to determine significant correlations between preserved expression of Ep-ICD/ EpEx, clinical parameters and prognosis of PCa patients. The correlation of Ep-ICD/ EpEx expression with patient survival (disease free survival, DFS) was evaluated using life tables constructed from survival data with Kaplan-Meier plots as described earlier [32]. Multivariate analysis was carried out using Cox regression models to determine the performance of preserved expression of EpEx/Ep-ICD as a marker in comparison with other clinical and pathological prognostic parameters including age, Gleason Score, AJCC stage, clinical risk (based on PSA), and grade in PCa patients. Bootstrap analysis was done to show an internal validation of data (S1 and S2 Tables).

## Results

### Immunohistochemical analysis of Ep-ICD and EpEx expression in prostate tissues

**Fig 2:**
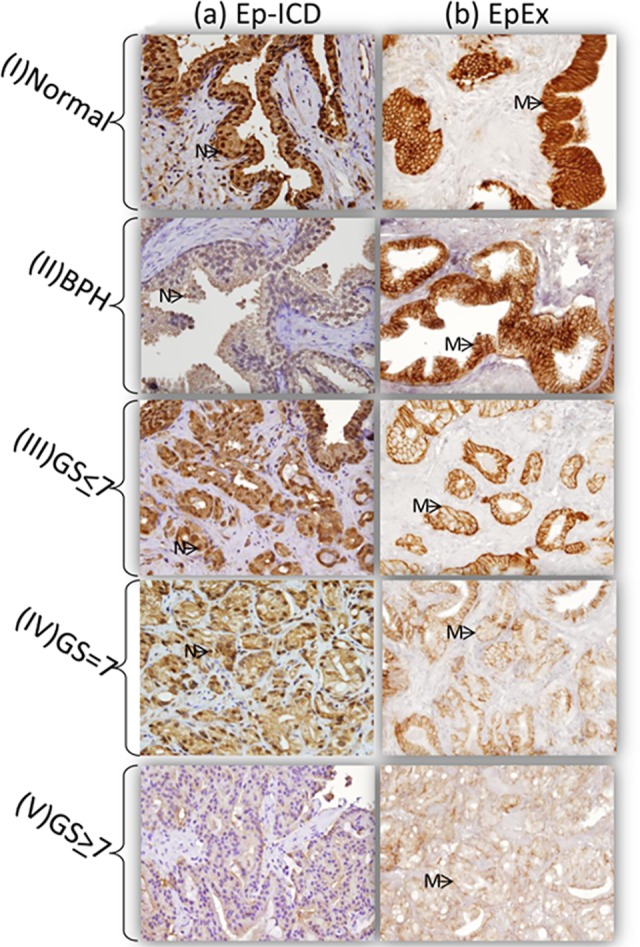
Immunohistochemical analysis of Ep-ICD and EpEx in prostate tissues. Panel shows (a) Nuclear/Cytoplasmic Ep-ICD immunostaining in (I) Normal, (II) BPH, and Prostate Adenocarcinoma: (III) Gleason Score < 7, (IV) Gleason Score = 7; and (V) Gleason Score > 7. Panel (b) Membrane EpEx immunostaining in (I) Normal (II) BPH, and Prostate Adenocarcinoma (III) Gleason Score < 7, (IV) Gleason Score = 7; and (V) Gleason Score > 7. Arrows show nuclear (N) Ep-ICD staining and Membrane (M) EpEx staining (Original Magnification x 400).

Representative photomicrographs depicting the subcellular localization of Ep-ICD and EpEx in normal prostate tissue, BPH and PCa of different GS are shown in Fig. 2. The distribution of IHC scores for (a) nuclear Ep-ICD, (b) cytoplasmic Ep-ICD, and (c) membranous EpEx, in normal, benign prostate hyperplasia (BPH), prostate intraepithelial neoplasia (PIN), and PCa tissues with respect to GS is shown in S1 Fig. Among the normal prostate tissues, 99 of 100 (99%), 100 of 100 (100%) and 81 of 100 (81%) expressed strong nuclear Ep-ICD, cytoplasmic Ep-ICD and EpEx membrane respectively (Table 1), while the PCa showed significant decrease in expression of these proteins (84/249, 33.7%; 240/249, 96.4%; and 142/249, 57.0% respectively; Table 1). BPH showed similar decrease in most of these in comparison with normal prostate tissues, though the decrease was smaller than in PCa (Table 1). Among PCa, there was a decrease in membranous EpEx with increasing AJCC staging and increasing GS (Table 1). Patients who had recurrence showed reduced nuclear Ep-ICD (p = 0.002) and decrease in membranous EpEx (p < 0.001) (Table 1). Comparison of PCa with normal tissues showed nuclear Ep-ICD expression had the highest specificity (99%) and sensitivity (66%) with an area under the curve (AUC) of 0.909 (Table 2). For comparison of BPH with cancer, nuclear Ep-ICD showed sensitivity (51%), specificity (66%) and AUC of 0.656.

**Table 1:** Analysis of Ep-ICD and EpEx expression in BPH and prostate cancer and correlation with clinic-pathological parameters.

Parameters	Total no. of cases	Ep-ICD (Nuc)		p-value	Odd’s ratio (95% C.I.)	Ep-ICD (Cyto)		p-value	Odd’s ratio (95% C.I.)	EpEx Membrane		p-value	Odd’s ratio (95% C.I.)
	(N)	n	(%)			N	(%)			n	(%)		
**Histology**													
Normal	**100**	**99**	**99**	**<0.001**	**0.01(0.001–0.1)^**1**^**	**100**	**100**	**<0.001**	**1.2(1.1–1.3)^**1**^**	81	81	0.518	0.8(0.4–1.6)^1^
BPH*	**83**	**42**	**50.6**	**0.006^**2**^**	**0.50(0.30–0.82)^**2**^**	**70**	**84.3**	**<0.001^**2**^**	**4.95(2.03–12.07)^2^**	64	77.1	0.001^**2**^	0.39(0.22–0.70)^**2**^
PIN**	**8**	**5**	**62.5**			7	87.5			**4**	**50**		
Cancer	**249**	**84**	**33.7**	**<0.001^**3**^**	**0.01(0.001–0.38)^**3**^**	240	96.4	0.054^**3**^	1.4(1.3–1.5)^3^	**142**	**57.0**	**<0.001^**3**^**	**0.3(0.2–0.5)^**3**^**
**Age**													
<65 yrs	126	45	35.7			121	96.0			73	57.9		
> 65 yrs	123	39	31.7	0.504	0.8(0.5–1.4)	119	96.7	0.762	1.2(0.3–4.7)	69	56.1	0.769	0.9(0.6–1.5)
**AJCC Stage**													
I	76	29	38.2			**74**	**97.4**			**54**	**71.0**		
II	158	53	33.5			**152**	**96.2**			**80**	**50.6**		
III	12	2	16.7			**12**	**100**			**6**	**50.0**		
IV	3	0	0	0.298	—	**2**	**66.7**	**0.041**	—	**2**	**66.7**	**0.028**	—
**Gleason**													
<7	108	34	31.5			105	97.2			**71**	**65.7**		
7	101	41	40.6			98	98.0			**61**	**60.4**		
>7	40	9	22.5	0.099	—	37	92.5	0.355	—	**10**	**25.0**	**<0.001**	—
**RISK^#^**													
Low	157	54	34.4			151	96.2			91	58.0		
Int.	50	18	36.0			48	96.0			27	54.0		
High	20	4	22.0	0.400	—	19	95.0	0.979	—	10	50.0	0.739	—
**Recurrence**													
No	**159**	**65**	**40.9**			154	96.9			**105**	**66.0**		
Yes	**90**	**19**	**21.1**	**0.002**	**0.4(0.2–0.7)**	86	95.6	0.598	0.7(0.2–2.7)	**37**	**41.1**	**<0.001**	**0.4(0.2–0.6)**

^1^Normal vs BPH,

^2^BPH vs Cancer,

^3^Normal vs Cancer

*BPH-Benign Prostatic Hyperplasia,

**PIN—Prostatic Intra-epthelial Neoplasia,

^#^RISK as defined by AJCC, data not available for 22 patients

**Table 2:** Biomarker analysis of Ep-ICD and EpEx in benign prostate hyperplasia and prostate cancer.

Normal Vs Cancer	Cut-off value	Sensitivity	Specificity	Area-Under-the-curve (AUC)	Sig.
Ep-ICD (Nuclear)	≥ 4	0.99	0.66	0.909	<0.001
Ep-ICD (Cytoplasm)	≥ 4	1.00	0.04	0.512	0.720
Membrane EpEx	≥ 3	0.81	0.43	0.630	<0.001
**BPH vs Cancer**					
Ep-ICD (Nuclear)	≥ 4	0.51	0.66	0.656	<0.001
Ep-ICD (Cytoplasm)	≥ 4	0.84	0.04	0.369	<0.001
Membrane EpEx	≥ 3	0.77	0.43	0.616	0.001

## Ep-ICD and EpEx as prognostic markers

**Fig 3:**
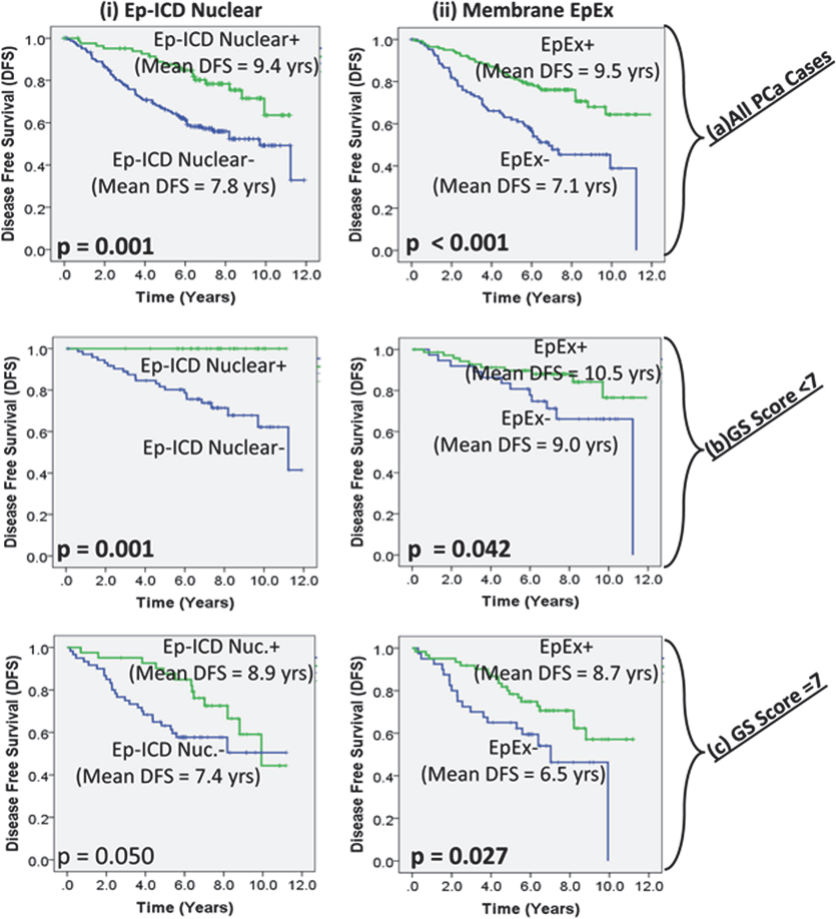
Kaplan Meier survival analysis of Ep-ICD and EpEx in prostate cancer patients. Kaplan-Maier analysis was performed dividing the patients as biomarker (Ep-ICD nuclear, Membrane EpEx) positive (green) and negative (blue). These were then further stratified by Gleason score. All the differences were significant and p-values are reported in figure as well as Table 2.

In Kaplan Meier analysis for all PCa (all GS taken together), patients with preserved expression of nuclear Ep-ICD had a mean DFS of 9.5 years, while those with reduced expression had mean DFS of 7.8 years (p = 0.001); patients with increased membrane EpEx had mean DFS of 9.5 years while those showing loss of membrane EpEx had mean DFS of 7.1 years, p < 0.001; Fig. 3). For PCa patients with GS less than 7, nuclear Ep-ICD (mean DFS could not be determined due to all cases being censored for increased nuclear expression) and membrane EpEx (increased expression mean DFS = 10.5 years, reduced expression mean DFS = 9.0 years) were associated with DFS (Fig. 3, p = 0.001, p = 0.042 respectively). Patients with GS of 7 showed significant association of nuclear Ep-ICD (increased expression mean DFS = 8.9 years, reduced expression mean DFS = 7.4 years) and membranous EpEx (increased expression mean DFS = 8.7 years, reduced expression mean DFS = 6.5 years) with survival (Fig. 3, p = 0.05 and p = 0.027 respectively). However, none of these proteins were associated with disease prognosis in PCa with GS greater than 7.

In Cox regression multivariate analysis for all PCa where nuclear Ep-ICD, risk, stage, membrane EpEx, and GS were included in the model, membrane EpEx, GS and nuclear Ep-ICD emerged as the most significant factors (p < 0.001, Hazard ratio (HR) = 0.294; p < 0.001, HR = 5.158; and p < 0.001, HR = 0.359 respectively, Table 3). In the same model including age and excluding risk patients with GS < 7, nuclear Ep-ICD emerged as the most significant factor (p = 0.05, HR = 0.025). For patients with GS of 7 in the same model AJCC stage (p = 0.001, HR = 6.214) and membranous EpEx (p = 0.019, HR = 0.452) emerged as the most significant factors for DFS (Table 3). None of these factors significantly correlated with prognosis for patients with GS greater than 7 in multivariate analysis.

**Table 3:** Univariate and multivariate analyses with respect to Gleason Score groups.

	Kaplan Meier Survival Analysis (p-value)	Multivariate Cox Regression Analysis (p-value)	Hazard Ratio (HR)	95% CI	
				Lower	Upper
**All Gleason Score**					
AJCC Stage	<0.001	----	----	----	----
Gleason Score	<0.001	<0.001	5.158	2.887	9.216
RISK	0.038	----	----	----	----
Ep-ICD Nuc	0.002	<0.001	0.359	0.205	0.631
EpEx Membrane	<0.001	<0.001	0.294	0.181	0.478
**Gleason Score <7**					
Age	0.018	----	----	----	----
Ep-ICD Nuc	0.001	0.05	0.025	0.001	1.038
EpEx Membrane	0.042	----	----	----	----
**Gleason Score = 7**					
AJCC Stage	0.016	0.001	6.214	2.057	18.267
Ep-ICD Nuc	0.050	----	----	----	----
Ep-ICD Cyto	0.015	----	----	----	----
EpEx Membrane	0.027	0.019	0.452	0.233	0.879

## Discussion

Prostate cancer management has come a long way in helping patients overcome cancer recurrence. In this study we have provided evidence that PCa patients having an increase in nuclear Ep-ICD and membranous EpEx (across all GS) were found to have better survival. Notably, for patients with GS less than 7, preserved expression of nuclear Ep-ICD emerged as the most significant marker in multivariate analysis for prolonged disease free survival, where these patients did not have any recurrence during the follow up period and patient’s with reduced nuclear Ep-ICD expression were at high risk of having aggressive disease. This is a key finding for identifying PCa patients with GS less than 7 (often thought to be indolent) that may end up having an aggressive cancer based on reduced nuclear Ep-ICD expression. Patients who are diagnosed GS less than 7 are most likely to get active surveillance as part of their clinical treatment. A subset of these patients at a later date will have progression of their cancer, but current diagnostic methods are inadequate to identify this subset of patients. As a part of the active surveillance for these patients, all of them will have 12 core biopsies and continued follow-up till they show evidence of cancer progression. Nuclear Ep-ICD expression in patients who are at low risk can identify those who actually need active surveillance and those who need active treatment. This will in turn reduce psychosocial burden of living with an indolent cancer without active treatment by telling patients, with more evidence, that their cancer is truly indolent. Sometimes patients that are on active surveillance with yearly biopsies will have an increased risk of erectile dysfunction [34]. These potential hazards can be avoided if patients, who are positive for nuclear Ep-ICD, can avoid active surveillance and thus not worry about cancer progression and other stressful clinical follow-up.

Several studies investigated expression of the full length EpCAM protein in PCa and tumor stroma [6,17,35,36], however none of these studies have correlated the expression of this protein with GS. Additionally, these studies did not report any significant correlation between EpCAM expression and disease progression, metastasis and survival. A more recent study by Benko et al. [37] described a correlation between EpCAM expression and poor prognosis of PCa, suggesting that EpCAM expression was a significant predictor of shortened biochemical recurrence free survival in clinically localized disease. However, these studies did not investigate the expression of EpCAM components but were limited to full-length EpCAM expression. Recent studies in other human cancers reported loss of membranous expression of the intracellular domain of EpCAM to be a frequent event and predicted poor survival in patients with pancreatic cancer and gastric cancer [38–40].

Our earlier preliminary report on Ep-ICD and EpEx expression analysis in ten epithelial cancers is the only study to our knowledge to-date describing Ep-ICD expression in PCa where in nuclear and cytoplasmic Ep-ICD immunopositivity was observed in 40 of 49 tumors; nuclear Ep-ICD was observed in 2 of 9 and cytoplasmic Ep-ICD in 1 of 9 normal prostate tissues [31]. Our recent results in an independent larger cohort showed nuclear Ep-ICD immunopositivity in the majority of normal prostate tissues, while 33.7% PCa were nuclear Ep-ICD immunopositive. This difference can be attributed to the fact that there was a larger cohort of both prostate normal as well as cancers in the current study. Moreover, the previous study used tissue microarrays (TMA) whereas our recent study used whole mount sections. The availability of whole mount sections in our current study allowed for a more thorough analysis of the tumors and their microenvironment. In some cases, Ep-ICD staining was localized to a sub-region of the normal tissue field and may not have been easily identified in small TMA specimens due to sampling effects. This may account for the lower nuclear Ep-ICD positivity in normal prostate tissues in our previous study. Moreover, the normal prostate tissues in TMA used previously were adjacent to the cancers, while in the current study we selected normal prostate cases which had a negative biopsy and continued to have negative biopsy for at least 5 years. Alternatively, the differences may be due to the small sample size (9 cases) or may be attributed to field cancerization effect observed in epithelial cancers, whereby the non-malignant tissue adjacent to the tumor might also show similar molecular alterations [41,42]. Furthermore, the previous TMA mainly consisted of stage 3 PCa most of which were high grade; our current study included tumors of all stages and all grades. Lastly, our previous study did not investigate the clinical or prognostic significance of Ep-ICD in these cancers.

Our current study showed expression of Ep-ICD (nuclear 33.7%, cytoplasmic 96.4%) and EpEx (57%) in PCa; however, higher expression was observed in normal prostate tissues analyzed (Ep-ICD nuclear 99%, Ep-ICD cytoplasmic 100%, membrane EpEx 81%). In comparison, other previous reports either did not investigate EpCAM expression in the normal prostate tissue [17,36], or some studies reported low EpCAM membranous expression in the adjacent benign prostate tissues [35,37]. The high Ep-ICD expression observed in normal prostate tissues in our study may be attributed to the use of domain specific antibody for Ep-ICD while the other studies used antibodies against the external domain of the full length EpCAM protein or it might be attributed to the selection of normal tissues that included cases which had a negative biopsy and continued to have negative biopsy for at least 5 years and not the adjacent benign prostate tissues obtained from cancer patients. Interestingly, in support of our findings of reduced nuclear Ep-ICD in prostate adenocarcinoma, a recent study in pancreatic cancer also reported reduced nuclear Ep-ICD staining in adenocarcinomas compared to other epithelial tumors [38]. In our study, nuclear Ep-ICD could differentiate between normal and cancerous prostate tissues with a high AUC (0.909), sensitivity (99%) and specificity (66%). Pathologists often have a challenge distinguishing a cancerous prostate biopsy from non-cancerous [43,44]. This leads to overtreatment of patients’ distress and side effects. About 23% of prostate biopsies are diagnosed as suspicious; 40% of these patients usually progress to cancer, therefore they require repeat biopsies which cause discomfort to the patients [45]. Some of the options for patients who have a “suspicious for cancer” diagnosis include: (i) a repeat biopsy; (ii) perform IHC for other biomarkers; (iii) send slides to a more experienced pathologist; or (iv) look at deeper tissue sections. Even with these options, the diagnosis of uncertainty may still exist. The combination of nuclear Ep-ICD scores with the pathological report may have the potential to distinguish, with confidence, a patient who is truly negative for the cancer because of the high sensitivity (99%) of this marker. Future studies are warranted to establish the clinical utility of nuclear Ep-ICD as a diagnostic marker for prostate biopsies that are “suspicious for cancer”.

One limitation of our study is the inability to explain reduced nuclear Ep-ICD expression in some PCa in comparison with the normal prostate tissues, while the cytoplasmic Ep-ICD expression remains consistent across the normal and cancerous prostate tissues, and the EpEx expression reduced in cancer. This might be regulated by intramembrane proteolytic cleavage mechanism that causes shedding of EpEx and translocation of the cytoplasmic domain in the cytoplasm, binding to FHL2 and β-catenin and translocation into the nucleus (Ep-ICD) in colon cancer cells [30]. However, in our study we observed reduction in nuclear Ep-ICD that correlated with poor disease prognosis, while the cytoplasmic Ep-ICD remained unaltered. We propose that further studies are needed to investigate the mechanism that prevents nuclear translocation of Ep-ICD in PCa. One possible explanation of cytoplasmic accumulation of Ep-ICD observed in PCa in our study is provided by a recent study which demonstrated the association between EpCAM and the proteolytic enzyme ERAP2, and described that EpCAM gets cleaved by ERAP2 in the cytoplasm which might explain the increased expression of Ep-ICD in the cytoplasm observed in our study [46]. Taken together these studies suggest that subcellular nuclear and cytoplasmic localization of Ep-ICD may be dependent on the cancer type and warrant in-depth studies in different cancers to explore their clinical utility both as prognostic markers and as predictors of response to EpCAM directed therapies.

There are other genomics based biomarkers that have been proposed to predict poor prognosis in PCa namely, PSCA, EZH2, TMPRSS2-ERG, E-cadherin, N-cadherin, ZEB1, and RASSF1A [47–52]. A major limitation of these genomic markers is their inability to accurately portray alterations in the cell attributed to changes at the transcriptional and translational (RNA and protein) levels in cancer cells. In comparison the protein based markers such as Ep-ICD overcome this lacuna. Furthermore, there are other biomarkers such as nurr1, OCT4, GRM1, HAI-1, SOX4, which are indicative of poor prognosis [53–57]. However, none of these biomarkers have the ability to stratify patients on the basis of risk i.e. they fail to predict which low risk patients would continue to be low risk and not have any progression of the cancer and which ones would have a recurrence.

In conclusion the most important finding of our study is the evidence in support of prognostic significance of nuclear Ep-ICD in stratifying PCa with GS less than 7 that are at high risk of disease recurrence for more rigorous follow up and management. There are times when patients who had a GS less than 7 ended up having a recurrence even though the traditional system of grading has deemed it to be at a low risk for disease recurrence. We propose that patients who have GS less than 7 and are nuclear Ep-ICD negative warrant more rigorous follow up and managed appropriately. Furthermore, analysis of nuclear Ep-ICD expression may also help avoid overtreatment of patients at low risk of disease recurrence, thereby reducing harmful side effects of therapy as well as reduce the economic burden on health care providers.

## Supporting Information

S1 Fig Ep-ICD and EpEx immunostaining score distribution in prostate normal, benign prostatic hyperplasia, prostate intra-epithelial neoplasia and prostate cancer tissues. All prostate tissue sections used for Ep-ICD / EpEx immunostaining were scored on the basis of % positivity and intensity. The total score was calculated as sum total of scores for % positivity and intensity as described in Materials and Methods. **S1a** shows the scatter plots depicting the score distribution of Ep-ICD and EpEx expression in prostate normal, benign prostatic hyperplasia, prostate intra-epithelial neoplasia and prostate cancer tissues. **S1b** shows box plot analysis of Ep-ICD and EpEx expression in prostate cancers with respect to Gleason’s Score.
(TIF)

S1 Table Internal Validation for risk assessment through bootstrap method. (DOCX)

S2 Table Bootstrap validation for cox multivariate. (DOCX)
