# Supplementary figures and images for: Nuclear Ep-ICD Expression Is a Predictor of Poor Prognosis in “Low Risk” Prostate Adenocarcinomas
Source: PLoS One. 2015 Feb 19;10(2):e0107586. doi: 10.1371/journal.pone.0107586 (PMC4335027; doi:10.1371/journal.pone.0107586)

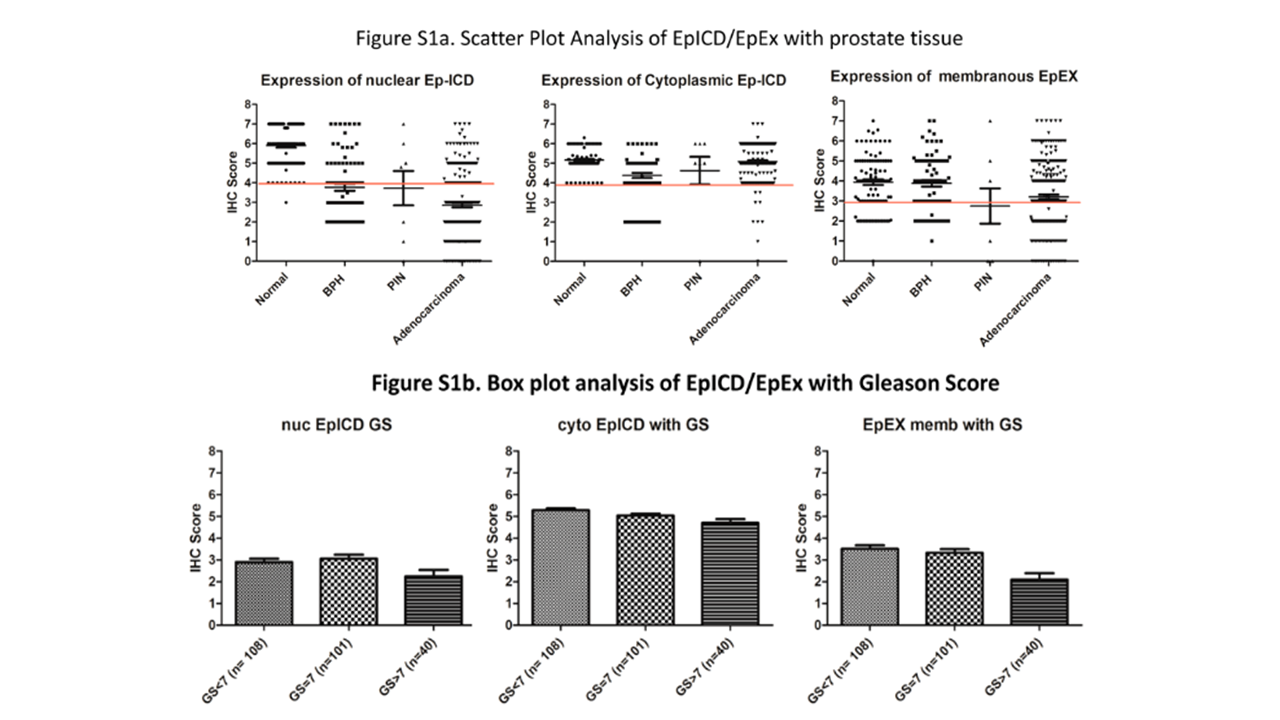

Supplement: S1 Fig — All prostate tissue sections used for Ep-ICD / EpEx immunostaining were scored on the basis of % positivity and intensity. The total score was calculated as sum total of scores for % positivity and intensity as described in Materials and Methods. S1a shows the scatter plots depicting the score distribution of Ep-ICD and EpEx expression in prostate normal, benign prostatic hyperplasia, prostate intra-epithelial neoplasia and prostate cancer tissues. S1b shows box plot analysis of Ep-ICD and EpEx expression in prostate cancers with respect to Gleason’s Score. (TIF) [file pone.0107586.s001.tif]
