# Supplementary material for: Nuclear Ep-ICD Expression Is a Predictor of Poor Prognosis in “Low Risk” Prostate Adenocarcinomas
Source: PLoS One. 2015 Feb 19;10(2):e0107586. doi: 10.1371/journal.pone.0107586 (PMC4335027; doi:10.1371/journal.pone.0107586)
Supplement: S1 Table — (DOCX) [file pone.0107586.s002.docx]

| **Parameters** | **Total no. of cases** | **Ep-ICD**  **(Nuc)** | | **Ep-ICD**  **(Cyto)** | | **EpEx**  **Membrane** | |
| --- | --- | --- | --- | --- | --- | --- | --- |
|  | (N) | Bias | Standard Error (95% C.I.) | Bias | Standard Error (95% C.I.) | Bias | Standard Error (95% C.I.) |
| **Histology** |  |  |  |  |  |  |  |
| Normal | 100 |  |  |  |  |  |  |
| BPH* | 83 | 0.006 | 0.10(0.01-0.44) | 0.017 | 0.22(2.01-2.94) | 0.580 | 0.35(0.36-1.59) |
| PIN** | 8 |  |  |  |  |  |  |
| Cancer | 249 | 0.003 | 0.01(0.00-0.02) | -0.002 | 0.05(1.32-1.52) | 0.006 | 0.09(0.17-0.53) |
| **Age** |  |  |  |  |  |  |  |
| <65 yrs | 126 |  |  |  |  |  |  |
| > 65 yrs | 123 | 0.170 | 0.23(0.49-1.36) | 0.452 | 1.45(0.28-6.12) | 0.024 | 0.26(.56-1.58) |
| **AJCC Stage** |  |  |  |  |  |  |  |
| I | 76 |  |  |  |  |  |  |
| II | 158 |  |  |  |  |  |  |
| III | 12 |  |  |  |  |  |  |
| IV | 3 | 0.128 | 0.20(0.10-0.87) | -0.120 | 0.27(0.08-1.08) | 0.081 | 0.45(0.11-1.7) |
| **Gleason** |  |  |  |  |  |  |  |
| <7 | 108 |  |  |  |  |  |  |
| 7 | 101 |  |  |  |  |  |  |
| >7 | 40 |  | --- | --- | --- | --- | --- |
| **RISK^#^** |  |  |  |  |  |  |  |
| Low | 157 |  |  |  |  |  |  |
| Int. | 50 |  |  |  |  |  |  |
| High | 20 |  | --- | --- | --- | --- | --- |
| **Recurrence** |  |  |  |  |  |  |  |
| No | 159 |  |  |  |  |  |  |
| Yes | 90 | 0.012 | 0.13(0.19-0.69) | 0.234 | 0.82(0.14-3.28) | 0.170 | 0.11(0.21-0.62) |

Table S1: Internal Validation for risk assessment through bootstrap method
