# Supplementary material for: Nuclear Ep-ICD Expression Is a Predictor of Poor Prognosis in “Low Risk” Prostate Adenocarcinomas
Source: PLoS One. 2015 Feb 19;10(2):e0107586. doi: 10.1371/journal.pone.0107586 (PMC4335027; doi:10.1371/journal.pone.0107586)
Supplement: S2 Table — (DOCX) [file pone.0107586.s003.docx]

|  | **Bias** | **Std. error** | **Sig.** | **95% CI** | |
| --- | --- | --- | --- | --- | --- |
|  |  |  |  | **lower** | **Upper** |
| **All Gleason Score** |  |  |  |  |  |
| AJCC Stage | 0.166 | 0.743 | .390 | -0.616 | 2.363 |
| Gleason Score | 0.054 | 0.321 | .002 | 1.035 | 2.273 |
| RISK | 0.066 | 0.505 | .896 | -1.209 | 0.820 |
| Ep-ICD Nuc | -0.045 | 0.297 | 0.003 | -1.615 | -0.445 |
| EpEx Membrane | -0.045 | 0.287 | 0.002 | -1.814 | -0.709 |
|  | | | | | |
| **Gleason Score <7** |  |  |  |  |  |
| Age | -0.288 | 1.626 | 0.021 | -2.859 | -0.228 |
| Ep-ICD Nuc | 0.019 | 0.271 | 0.001 | -13.006 | -11.913 |
| EpEx Membrane | 0.000 | 0.470 | 0.104 | -1.670 | 0.220 |
|  | | | | | |
| **Gleason Score =7** |  |  |  |  |  |
| AJCC Stage | -0.325 | 2.715 | 0.203 | -1.336 | 3.454 |
| Ep-ICD Nuc | 0.256 | 0.611 | 0.223 | -1.880 | 0.000 |
| Ep-ICD Cyto | 1.134 | 3.805 | 0.122 | -3.445 | 12.563 |
| EpEx Membrane | -0.325 | 2.715 | 0.203 | -1.336 | 3.454 |

**Table S2**. Bootstrap validation for cox multivariate
